# A randomised trial to assess fluid and electrolyte balance responses following ingestion of different beverages in young and older men

**DOI:** 10.1007/s00421-023-05241-0

**Published:** 2023-06-09

**Authors:** Nidia Rodriguez-Sanchez, Stuart D. R. Galloway

**Affiliations:** Physiology, Exercise and Nutrition Research Group, Faculty of Health Sciences and Sport, University of Stirling, Stirling, FK9 4LA UK

**Keywords:** Hydration, Dehydration, Macronutrients, Renal excretion, Urine, Creatinine

## Abstract

**Background:**

Older adults are susceptible to dehydration and fluid overload due to a reduced ability to maintain homeostatic control of fluid and electrolyte balance.

**Purpose:**

To assess fluid and electrolyte balance responses in young and older men following ingestion of commonly consumed beverages differing in composition.

**Methods:**

12 young and 11 older men were recruited. Euhydrated body mass was recorded. Participants consumed 1L (250 ml every 15 min) of water, fruit juice, a sports drink or low-fat milk in a randomized cross-over design. Urine and blood samples were obtained before and after the drinking period and every hour thereafter for 3-h. Samples were used to determine osmolality, electrolytes (Na^+^ and K^+^), water clearance, and glomerular filtration rate.

**Results:**

Free water clearance was significantly higher in Young than Older at 1 and 2 h after the ingestion of W and S (p < 0.05). Net Na^+^ and K^+^ balance were not different between Young and Older (p = 0.91 and p = 0.65) adults, respectively. At 3 h Na^+^ balance was negative after ingesting water and fruit juice, but neutral after sport drink and milk. Net K^+^ balance was neutral at 3 h after ingesting milk, but negative after water, fruit juice and sport drink.

**Conclusions:**

Milk was retained longer than other beverages in Young, but not in Older, despite similar net electrolyte balance responses. Older had higher fluid retention in the first 2 h after the ingestion of all beverages, except for milk when compared to Young, indicating an age-related loss of ability to regulate fluid balance under current study conditions.

**Supplementary Information:**

The online version contains supplementary material available at 10.1007/s00421-023-05241-0.

## Introduction

Ageing is accompanied with changes in the homeostatic control systems that regulate fluid and electrolyte balance (Rolls and Philips 1990) increasing the risk of older adults becoming dehydrated, but also predisposing them to developing problems of fluid overload (Allison and Lobo 2004). Inadequate hydration status has been linked to urinary tract infections, impaired cognitive function, and delirium, and is related to a higher incidence of falls in older adults (Rowe and Juthani-Mehta 2013; Dubeau 2006). Whereas an impaired ability to excrete excess fluid promptly due to an age-related reduction in glomerular filtration rate [GFR (Dontas et al. 1972)] can lead to passive reabsorption of fluid with a higher risk of presenting water overload and hyponatraemia (Musso and Oreopoulos 2011). The consequences of overhydration can be life-threatening (El-Sharkawy et al. 2014). Of course, any impact of ageing on human health or function is complicated by the interaction of various factors including lifestyle choices, such as physical activity behaviours. For example, research on musculoskeletal function is often complicated by the interaction between ageing and sedentary behaviour (Moreno-Agostino et al. 2020, Paterson and Warburton 2010).

However, under free-living conditions, older adults are more prone to become dehydrated than their younger counterparts. Fluid deficits in older adults can occur through a variety of routes such as: a blunted thirst response, resulting from a decrease in the sensitivity of volume and osmoreceptors (Kenney and Chiu 2001); loss of lean mass leading to a reduction in total body water content by 10–15% (Allison and Lobo 2004, Adams et al. 2014); and/or reduced capacity of kidneys to concentrate urine (Sands 2012). Older adults also have a reduced capacity to handle sodium loads, making them more prone to over-expansion of the extracellular fluid compartment (Luckey and Parsa 2003). Ageing is associated with progressive tubular dysfunction, resulting in decreased sodium reabsorption and reduced potassium secretion (Musso and Oreopoulos 2011). These differences in electrolyte handling suggest that older adults cannot deal with sodium or potassium loads as efficiently as younger adults, making them prone to electrolyte abnormalities such as dysnatremias as older adults can experience a decrease in the ability to excrete or reabsorb sodium (Musso et al. 2006, Schlanger et al. 2010). Older adults have a decreased transtubular potassium gradient when compared with their young population (Musso and Jauregui 2014, Lindeman et al. 1985). Ageing confers a natural decline in several aspects of gastrointestinal physiology, including gastrointestinal peristalsis, enteric nervous system, intestinal mucosa, mucosal immunity, and gut absorption (Atillasoy and Holt 1993, Hooper et al. 2014; Russell 1992). The risk of dehydration in older adults is exacerbated by cognitive and behavioural changes such as a lack of knowledge, or misconceptions concerning the effects of drinking, or not drinking, sufficient fluid (Hooper et al. 2014, Bhanu et al. 2020) Furthermore, many older adults limit or avoid beverages to reduce the frequency of urination (Godfrey et al. 2012). All older adults are at risk of low-intake dehydration, and they should be encouraged to ingest an adequate amount of fluids (Volkert et al. 2019). Different beverages can contribute to the daily fluid intake. However, little is known about the renal handling of popular and commercially available beverages with different composition in the older adult population.

Data on young adults highlights that electrolyte composition, as well as certain macronutrients (i.e., protein/carbohydrate), are important in determining the fluid retention response to beverage ingestion (Maughan et al. 2016, 2019). However, to our knowledge, only a pair of companion papers from the same sample of older adults have investigated fluid and electrolyte balance responses to beverages with different macronutrient and electrolyte composition (Clarke 2019, Wolf et al. 2019). In their sample, an amino acid-based beverage (containing 5 amino acids, 30 mmol Na^+^ and 10 mmol K^+^) had the highest hydration potential compared to water in the older group at 2 h after ingestion. Therefore, we aimed to further investigate the fluid and electrolyte balance responses to a range of frequently consumed commercially available beverages differing in macronutrient and electrolyte content, and to compare responses between young and older adult males. We hypothesized that beverages with a higher macronutrient/electrolyte content would lead to greater fluid retention than water in young adults, but that these responses would be blunted in an older adult group.

## Methods

The fluid and electrolyte balance responses after ingestion of four commonly consumed and commercially available beverages were tested in young and older volunteers. The beverages were: still water (as control); fruit juice with a moderate carbohydrate and potassium content; a sport drink with moderate carbohydrate and sodium content (S); and a low fat 1% milk with moderate carbohydrate, protein, and sodium and potassium content. The test beverages were specifically chosen with an energy content in a range of 230-350 kcal/L (except for water) and provided either sodium, potassium, or a mix of electrolyte content (Table 2). The study was approved by the University of Stirling—School of Sport Research Ethics Committee (SSREC #753). All participants provided their informed consent for their participation.

### Pre-trial standardization/exclusion criteria

Twenty-four healthy active volunteers were recruited into two groups: n = 12 were allocated to the young group (18-35y) and n = 12 to the older group (> 55y) with participants matched for stature and body mass. One participant in the older group dropped out **(**Table 1**)**. Those participants with a history of cardiovascular, renal, musculoskeletal, or metabolic disease determined from a health screen questionnaire were excluded. Participants following an energy restricted diet and/or exercise plan to lose or gain mass were excluded. Volunteers were asked to record and replicate their food and fluid intake two days before each experimental trial, and to refrain from alcohol ingestion and vigorous physical activity for 24 h before all trials.

**Table 1:** Participant anthropometric characteristics, hydration habits, caffeine and alcohol intake, and self-reported physical activity

	Age group	
	Young (n = 12)	Old (n = 11)
Age (years)	24.5 (4.3)	63.8 (5.5)*
Body mass (kg)	76.7 (9.1)	78.2 (9.1)
Stature (m)	1.79 (0.07)	1.77 (0.06)
Body mass index (kg/m^2^)	24.0 (2.3)	25.1 (3.6)
Self-reported fluid intake (ml/d)	1929 (310)	1780 (215)
Caffeine intake (mg/d)	211.3 (112.7)	230.5 (79.9)
Weekly alcohol intake (units)	4.6 (5.7)	7.5 (3.1)
Moderate to vigorous physical activity (hours per week)	5.9 (1.0)	6.7 (2.6)
Participants reporting to exercise 4 or more times per week (n)	9	8

Values are mean (SD)

*Indicates significant difference between young and older groups. (p < 0.05)

### Participant characteristics on entry into the study

The physical activity levels of the participants varied, with the older group reporting an average of 6.7 h of moderate/intense exercise per week, compared to the young group's reported average of 5.9 h per week. Participants engaged in a range of sports or activities, with jogging more than 5 k, swimming more than 600 m, and gym combining strength and aerobic exercises being the most frequently reported in the young group. In contrast, the older group reported cycling, gym combining strength and aerobic exercises, and hill walking as their most frequently reported activities, with some participants also reporting engaging in less common activities such as ice skating and skydiving (Table 1).

### Experimental procedure

Participants attended the laboratory for four experimental trial days, each separated by 7-days. All trials were conducted in the morning after an overnight fast (> 8 h). Participants ingested 500 ml of still water (Highland Spring^®^) 1 h before attending the laboratory to ensure euhydration upon arrival. Euhydration was required to ensure that all participants could provide urine samples over the entire study monitoring period. When the participants arrived at the laboratory, they were asked to empty their bladder/bowel, urine was collected, urine mass was recorded, and a 5 ml aliquot retained for analysis. An intravenous cannula was inserted into an antecubital vein, and initial near nude body mass was recorded (in underwear only). Participants adopted a seated position for 15 min after which a baseline blood sample was drawn. Participants then consumed a fixed volume (1L, divided in four aliquots of 250 ml provided every 15 min) of water (Highland Spring®), fruit juice (Tropicana, Trop50^®^), sport drink (Lucozade Sport^®^) or milk (low fat 1% skimmed milk) (Table 2). Test beverages were administered in a randomised counterbalanced order. Standard commercial beverages were purchased as a single batch or from a single source (for products with a short shelf life) to be used for all trials. All beverages were stored at a standard refrigerated temperature (4–6 °C) until serving.

**Table 2:** Water, energy, macronutrient, sodium, and potassium content of tested beverages

Beverage	Water content (%)	Energy (kcal/ 100 ml)	CHO (g/100 ml)	Fat (g/100 ml)	Protein (g/100 ml)	Sodium (mmol)	Potassium (mmol)
Water–control	100	0	0.0	0.0	0.0	0	0
Fruit juice	95	23	4.4	0.0	0.3	0	28
Sport drink	94	28	6.4	0.0	0.0	22	3
Low fat 1% milk	91	35	5.0	0.1	3.4	19	23

Apart from water, test beverages were selected to provide similar carbohydrate (CHO) content, but with differing protein and electrolyte composition

Immediately at the end of the 60-min beverage ingestion period, participants were asked to empty their bladder, and subsequently each hour for the next 3 h to monitor urine volume. Participants could urinate at any time they required but they were also asked to empty their bladder completely every 60-min to track hourly urine production. If volunteers needed to urinate before the 60-min period, the urine was collected and added to the urine produced at the next 60-min time point. Urine mass was recorded to determine cumulative urine mass every 60-min and a 5 ml aliquot was retained for analysis. Blood samples were drawn immediately after the drinking period, and every 60-min for the next 3 h. All blood samples were drawn from participants after they had remained in a seated position for at least 15-min (Supplementary Appendix 1).

### Blood, serum, and urine analysis

Total urine mass (to nearest g) was measured over the 3 h post beverage ingestion. Samples of urine obtained each hour were analysed for urine osmolality, creatinine concentration and urine electrolyte content. From each urine void, a 5 ml aliquot was collected and stored at 4 °C for the analysis of urine osmolality, electrolyte content (sodium and potassium) and creatinine determination. Whole blood samples were dispensed into a serum tube for osmolality and electrolyte determinations, and an EDTA tube with plasma stored for later analysis of creatinine. Urine and serum osmolality were measured in duplicate using a freezing point depression method (Löser osmometer) on day of collection. Sodium and potassium concentration in urine and serum were measured in duplicate using flame-photometry (PFP7/C Clinical Flame Photometer, Jenway) within 5-days of collection.

Macronutrient data of test beverages were obtained from product nutrition information. Beverage electrolyte data were obtained by flame-photometry analysis.

### Data calculations and statistical analysis

All the participants achieved a positive net fluid balance of 1000(g) following the fixed volume of the fluid ingested (1L). Net fluid balance each hour was then calculated from the total mass of urine (cumulative urine output) that had been collected to that point. Electrolyte balance on each trial was determined by subtracting the amount of sodium or potassium excreted through urine from the total sodium or potassium ingested within the test beverage.

Urine creatinine was used to estimate GFR (Levey et al. 2009, Devanand and Chithrapavai 2013). Free water clearance was calculated at 1 h, 2 h and 3 h after test beverage ingestion following the equations described by Wolf et al. (Wolf et al. 2019).

Cumulative urine outputs, by beverage and between groups, were compared using repeated measures ANOVA. Participant characteristics were analysed by paired t-tests. Data for net fluid balance, and electrolytes were analysed by 3-way ANOVA (beverage x time point x age group) to identify within and between group differences. All statistical analyses were completed using a statistical software package (IBM SPSS Statistics, V.23). Statistical significance was accepted at *P* < 0.05. Data are presented as mean (95% CI) or mean (SD) in the text, and as mean (SD) in Tables and Figures.

## Results

Of the twenty-four participants recruited (12 young and 12 older men), n = 1 older adult participant dropped out of the study due to aversion with the blood sampling procedures. No participants experienced any adverse effect or gastrointestinal symptoms following beverage ingestion.

Mean urine and serum osmolality when participants arrived at the lab, prior to beverage ingestion, was not different between young and older adults, except for urine osmolality on trial M (Table 2). Mean serum and urine osmolality demonstrated that both groups began all trials in a euhydrated state.

### Cumulative urine output and urine osmolality responses to beverage ingestion

Urine mass did not differ between trials immediately after the beverage ingestion period for Young and Older. However, 1 h after the ingestion of water, the cumulative urine output for Young was significantly higher than Older. This was sustained at 2 h post drinking (p < 0.05). Urine osmolality was significantly lower in Young compared to Older at 1 h after ingestion of water; and significantly higher in Young than Older at 2 h after the ingestion of sport drink and milk, and at 3 h for water, fruit juice and sport drink (Table 2).

### Net fluid balance (NFB)

**Fig. 1:**
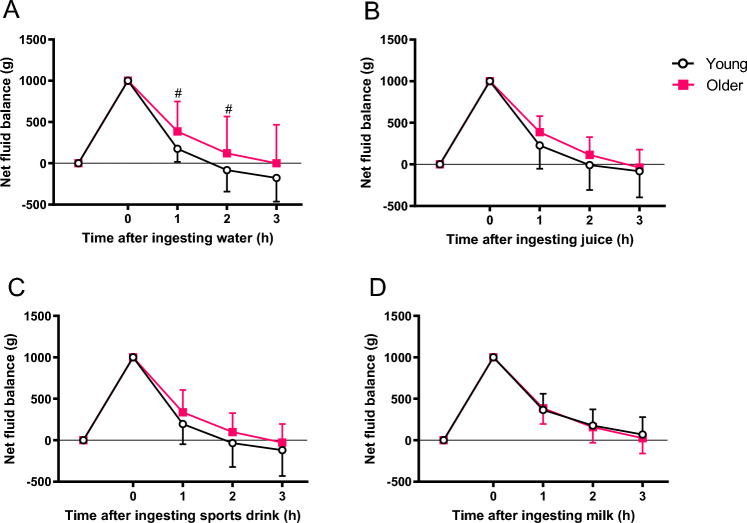
Net fluid balance following the ingestion 1L of water (**A**), fruit juice (**B**), sports drink (**C**), and milk (**D**) in young and in older men. Values are mean (SD). #p < 0.05 between age groups

At 1 and 2 h after ingesting water, there were significant differences between Young and Older. However, no significant difference was observed between age groups when comparing NFB at 3 h after ingestion of any of the different beverages. NFB after ingestion of milk was significantly different from water in Young, but not in Older (Fig. 1).

### Estimated GFR and free water clearance

**Fig. 2:**
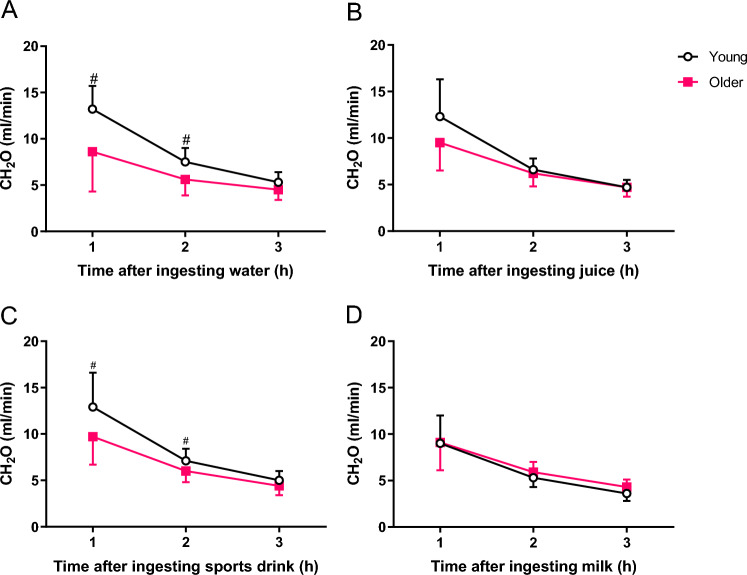
Free water clearance responses after the ingestion of 1 L of water (**A**), fruit juice (**B**), sport drink (**C**) and milk (**D**) at 1, 2 and 3 h after ingestion of test beverage in young and older men. Values are mean (SD). #p  < 0.05 between age groups

Mean (SD) estimated GFR was lower in older participants (74.1 mL/min/1.73 m^2^; 70.8, 77.5) when compared with the younger group (98.9 mL/min/1.73 m^2^; 94.8, 102.9) (p < 0.001). Estimated free water clearance rate was significantly higher in Young compared with Older, particularly at 1 h and 2 h (p < 0.05) after the ingestion of water and sport drink (Fig. 2). No other differences were observed at other time points between groups. When the test beverages were compared with water within the same age group, for young adults, milk was different from water at 1 h, 2 h, and 3 h post ingestion (p < 0.05), but this was not observed in the older adult group.

### Electrolyte balance

**Fig. 3:**
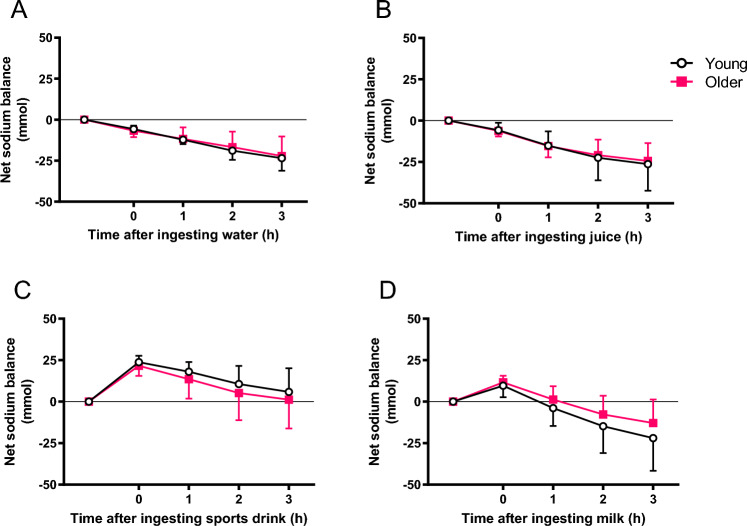
Net sodium balance after the ingestion of 1 L of water (**A**), fruit juice (**B**), sport drink (**C**) and milk (**D**) at 1, 2 and 3 h after ingestion of test beverage in young and older subjects. Values are mean (SD)

**Fig. 4:**
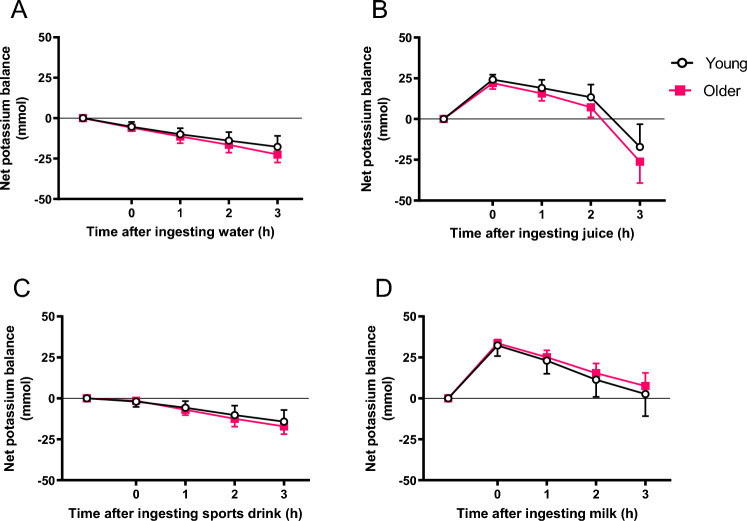
Net potassium balance after the ingestion of 1 L of water (**A**), fruit juice (**B**), sport drink (**C**) and milk (**D**) at 1, 2 and 3 h after ingestion of test beverage in young and older subjects. Values are mean (SD)

Net sodium balance 3 h after test beverage ingestion was greater in both age groups when they ingested sport drink, leading to a more positive sodium balance in comparison with water. No other significant differences in sodium balance were observed between test beverages or between groups (Fig. 3). Fruit juice led to a more positive potassium balance 2 h after its ingestion in both groups when compared with water (p < 0.05) (Fig. 4). There was no significant difference between age groups in sodium or potassium concentration in serum at any time point. However, in the young group only the sodium concentration in serum was significantly higher when participants drank sport drink than when they ingested water, at 2 h and 3 h after ingestion. In the older group, there was a significant difference in serum potassium at 1 h, 2 h, and 3 h after ingesting milk in comparison with water (Table 3).

**Table 3:** Cumulative urine mass, urine osmolality, serum osmolality and electrolytes (Na^+^ and K^+^) in serum prior and following the ingestion of the test beverages in young and older adult groups

	Age group	Test beverage	Time after ingestion of test beverage (min) (post ingestion time point)				
			− 60	0	60 (1 h)	120 (2 h)	180 (3 h)
Cumulative urine mass	Young	Water	–	–	825 (158)^+^	1082(261)^+^	1177 (288)
		Fruit Juice			773 (279)	1009 (303)	1082 (316)
		Sport drink			804 (243)	1034 (289)	1120 (311)
		Low fat 1% milk			635 (196)	822 (195)	956 (339)
	Old	Water	–	–	544 (285)^+^	791 (342)^+^	987 (331)
		Fruit Juice			599 (196)	878 (223)	1037 (227)
		Sport drink			610 (204)	861 (190)	994 (199)
		Low fat 1% milk			625 (196)	851 (184)	978 (174)
Urine osmolality (mOsmol/l)	Young	Water	545 (323)	276 (188)	86 (21)^+^	194 (103)	403 (149)^+^
		Fruit Juice	491 (305)	298 (260)	111 (34)	229 (94)	533 (165)^+^
		Sport drink	523 (316)	315 (237)	106 (52)	255 (139)^+^	519 (221)^+^
		Low fat 1% milk	578 (290)^+^	358 (267)	249 (159)	381 (104)^+^	566 (187)
	Old	Water	387 (122)	221 (88)	146 (54)^+^	161 (51)	222 (63)^+^
		Fruit Juice	352 (111)	250 (109)	139 (41)	171 (42)	251 (85) ^+^
		Sport drink	434 (210)	310 (183	111 (27)	158 (52)^+^	287 (123)^+^
		Low fat 1% milk	385 (139)^+^	256 (151)	174 (64)	268 (106)^+^	411 (160)
Serum osmolality (mOsmol/l)	Young	Water	295 (4)	295 (4)	294 (3)	295 (4)	295 (3)
		Fruit Juice	296 (3)	297 (3)	295 (3)	296 (3)	295 (3)
		Sport drink	296 (3)	297 (3)	296 (3)	295 (3)	295 (3)
		Low fat 1% milk	297 (3)	297 (2)	297 (3)	295 (2)	296 (2)
	Old	Water	296 (4)	294 (4)	293 (3)	294 (4)	295 (4)
		Fruit Juice	295 (3)	296 (4) *	294 (4)	294 (4)	295 (4)
		Sport drink	295 (3)	295 (3)	294 (3)	293 (3)	294 (3)
		Low fat 1% milk	294 (3)	296 (3)*	296 (3) *	295 (3)	295 (4)
Serum sodium (mmol)	Young	Water	142 (6)	140 (5)	141 (5)	139 (6)	139 (7)
		Fruit Juice	141 (5)	139 (4)	143 (4)	143 (5)	144 (5)
		Sport drink	144 (5)	142 (6)	143 (6)	147 (7)*	146 (6)*
		Low fat 1% milk	141 (7)	143 (6)	143 (6)	143 (6)	142 (5)
	Old	Water	142 (7)	139 (6)	139 (6)	139 (6)	142 (6)
		Fruit Juice	142 (6)	142 (6)	142 (7)	144 (5)	142 (5)
		Sport drink	141 (5)	143 (5)	143 (7)	144 (7)	142 (6)
		Low fat 1% milk	140 (6)	142 (8)	141 (8)	139 (6)	140 (7)
Serum potassium (mmol)	Young	Water	4.1 (0.7)	4.3 (0.6)	4.3 (0.6)	4.3 (0.5)	4.4 (0.6)
		Fruit Juice	4.1 (0.5)	4.2 (0.5)	4.4 (0.5)	4.4 (0.3)	4.5 (0.3)
		Sport drink	4.2 (0.5)	4.3 (0.6)	4.3 (0.7)	4.6 (0.8)	4.4 (0.5)
		Low fat 1% milk	4.1 (0.5)	4.3 (0.7)	4.5 (0.7)	4.6 (0.8)	4.4 (0.5)
	Old	Water	4.4 (0.5)	4.6 (0.5)	4.6 (0.5)	4.7 (0.5)	4.7 (0.5)
		Fruit Juice	4.6 (0.6)	4.7 (0.6)	4.7 (0.6)	4.8 (0.5)	4.8 (0.5)
		Sport drink	4.4 (0.4)	4.4 (0.5)	4.5 (0.6)	4.5 (0.5)	4.2 (0.5)
		Low fat 1% milk	4.5 (0.3)	4.9 (0.5)	5.0 (0.5)*	5.0 (0.4)*	5.0 (0.3)*

Values are mean (SD)

*Indicates significant difference from water within group and column

^+^Indicates significant difference between young and older groups. (p < 0.05)

## Discussion

We investigated fluid and electrolyte balance in young and older adults after the ingestion of beverages with different nutrient composition and assessed their retention over three hours post ingestion. In young adults, we replicated our previous observations (Maughan et al. 2016, 2019) by demonstrating that milk led to greater net fluid balance than water over the follow-up period. However, this effect of milk was not observed in older adults. Our findings suggest that when older adults ingest beverages differing in electrolyte and macronutrient content the resulting fluid delivery, absorption and electrolyte balance appears insufficient to impact upon overall net fluid balance, 3 h after ingestion. In the first 2 h after water ingestion, a lower free water clearance rate and more positive net fluid balance was observed in older adults. No differences in the electrolyte balance between age groups were observed. The different responses between older and young male adults are likely due to age-related changes in gastric emptying and absorption of fluid, declines in renal function.

### Gastric emptying and intestinal absorption of fluid

In the present study, the difference in fluid balance response between young and older adult groups may be influenced mostly by gastric emptying rates. In older adults an age-related reduction in gastric acid secretion would potentially allow for a greater gastric emptying rate of milk by reducing casein clot formation (Huppertz and Chi 2021). It has been demonstrated previously that basal and maximal gastric acid output both decrease in ageing humans (Holt et al. 1989). Dangin et al. (2003) observed that in young people casein could be classified as a slow protein due to reduced gastric emptying rate and subsequent amino acid delivery, when compared against whey protein. However, in older adults there is a faster casein-emptying rate due to an age-related decline in gastric acid secretion.

Most of the water (ingested and emptied into the gastrointestinal tract) is reabsorbed in the small intestine, and remaining water in the colon. The small intestine is highly permeable promoting a rapid balance of the digestive content through the absorption of electrolytes and nutrients (Leiper 1998). Water can pass through the intestinal epithelium via the paracellular or the transcellular route. The transcellular route consists of different mechanisms: passive diffusion, cotransport and the aquaporins (Laforenza 2012). To our knowledge, there are no studies documenting modifications of any of these mechanisms that could impair water bioavailability in older adults. However, the mucosal epithelium is also important for water and electrolyte absorption, and ageing impairs mucous secretion (Branca et al. 2019). Thus, it is not known if age-related changes in gastrointestinal physiology negatively impact upon water and electrolyte absorption (Luchette and Yelon 2017).

### Renal function

The ability to concentrate urine declines with age. In the Baltimore Longitudinal Study of Ageing, individuals aged 60–79 years had a ~ 20% reduction in maximal urine osmolality, a 50% decrease in the ability to reabsorb sodium and urea, and reduced capacity to concentrate solutes, when compared with 20–39 years old group (Lindeman et al. 1985, Rowe et al. 1976). In the present study, it was observed that there was a 39% lower urine osmolality in the older group 3 h after beverage ingestion, regardless of beverage composition ingested, when compared with the younger group. This finding reinforces the decline in urinary concentrating capacity with ageing (Musso et al. 2012).

In the present study, the volume overload induced by ingesting 1 L of fluid in 1 h, when in an already euhydrated state, leads to a more prolonged fluid retention of water in older adults, likely due to a reduced homeostatic control response compared to young adults (Beck 2000). This is evidenced by lower total urine output and calculated free water clearance in the first 2 h after ingestion of water in older than in younger adults. Further, it is known that GFR is preserved until about the age of 40 years, after that it declines linearly at an average rate of about 8 ml/min/decade (Silva 2005). In the present study, we observed that mean estimated GFR was 25.7 mL/min lower in older than in younger men, demonstrating a modest reduction in estimated GFR in the older group (6.5 mL/min per decade). Thus, it seems that differences between young and older adults, particularly in the initial response to water ingestion, relate to age related declines in homeostatic control and renal function that result in delayed excretion of the fluid overload.

### Electrolyte balance

Older people are prone to expansion of total body water when challenged with a volume or sodium overload. Older adults have been reported to have a diminished capacity for renal sodium excretion (Luckey and Parsa 2003, Luft et al. 1979, Stachenfeld et al. 1996). However, as a rapid increase in GFR is required to deal with acute salt overload it is natural to consider that older people (with a normally reduced GFR) will present a limited ability to manage sodium loads (Hall 2016). A reduced capacity to handle sodium loads would predispose older adults to overexpansion of the extracellular fluid compartment. In the present study, the commercially available beverages that were provided were in the range of 0–22 mmol/L sodium. This range is likely insufficient to challenge homeostatic controls and observe any significant difference between groups or between beverages. Indeed, no differences were noted in net sodium balance response to the sodium containing sport drink between young and older adults. The mean loss of sodium in urine appeared to be less in older than younger adults after milk ingestion; however, this did not reach statistical significance. Future research is needed to examine electrolyte balance responses in older adults following larger sodium loads. Potassium excretion is reported to be reduced in the aged kidney and is attributed to the combination of low potassium secretion (Musso et al. 2006) and high potassium reabsorption (Eaton and Pooler 2018). As a result, healthy older people have a reduction in the transtubular potassium gradient compared with younger adults, leading to reduced excretion of potassium load in older adults (Musso et al. 2006). In the present study no differences were observed between age groups suggesting no changes in handling of potassium across the age range and potassium loads examined.

## Conclusion

Further investigation is necessary to examine older adults under a fluid deficit condition to establish how older adults deal with fluid replacement and fluid overload situations. In addition, future research could aim to investigate more significant challenges to homeostasis using test drinks with greater electrolyte and macronutrient composition differences. However, under the conditions of the present study, it is evident that free water clearance is impaired in the first two hours following a fluid overload in older adults when compared with responses in young.

## Supplementary Information

Below is the link to the electronic supplementary material.

Supplementary file1 (DOCX 174 KB)

## Data Availability

The datasets generated during and/or analyzed during the current study are available from the corresponding author on reasonable request.

## Abbreviations

ANOVA: Analysis of variance
GFR: Glomerular filtration rate
NFB: Net fluid balance
