# Supplementary material for: A randomised trial to assess fluid and electrolyte balance responses following ingestion of different beverages in young and older men
Source: Eur J Appl Physiol. 2023 Jun 9;123(10):2331–40. doi: 10.1007/s00421-023-05241-0 (PMC10492686; doi:10.1007/s00421-023-05241-0)
Supplement: Supplementary file 1 — Supplementary file1 (DOCX 174 KB) [file 421_2023_5241_MOESM1_ESM.docx]

#### **Supplementary Material**

####
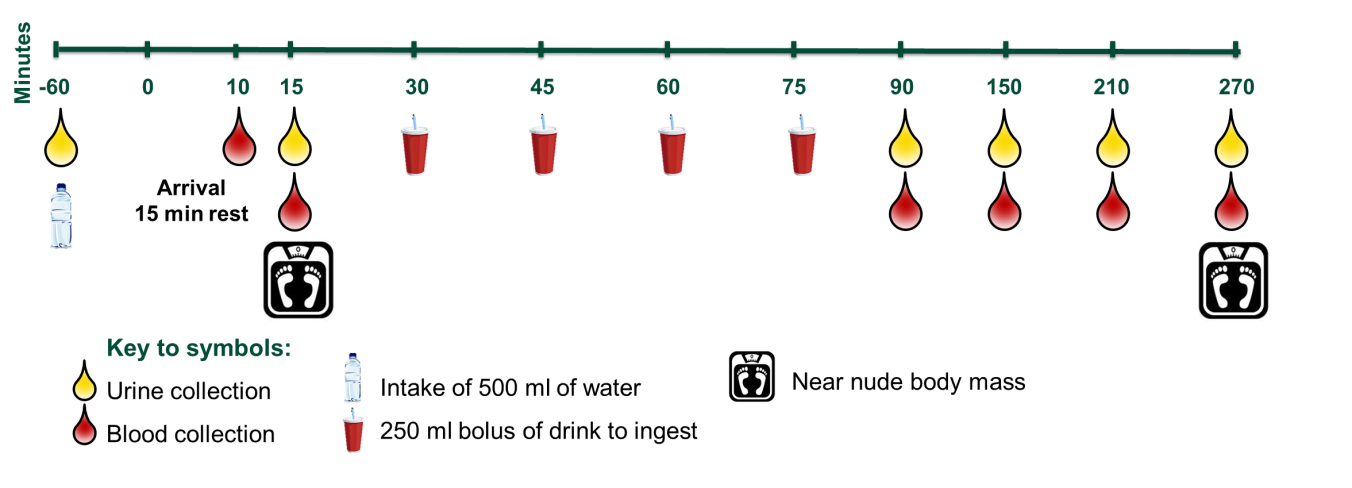


-90 -60 -25 -10 0 15 30 45 60 120 180 240

#### **Appendix 1.** Schematic of experimental trial days. All trials were conducted first thing in the morning after an overnight fast.
